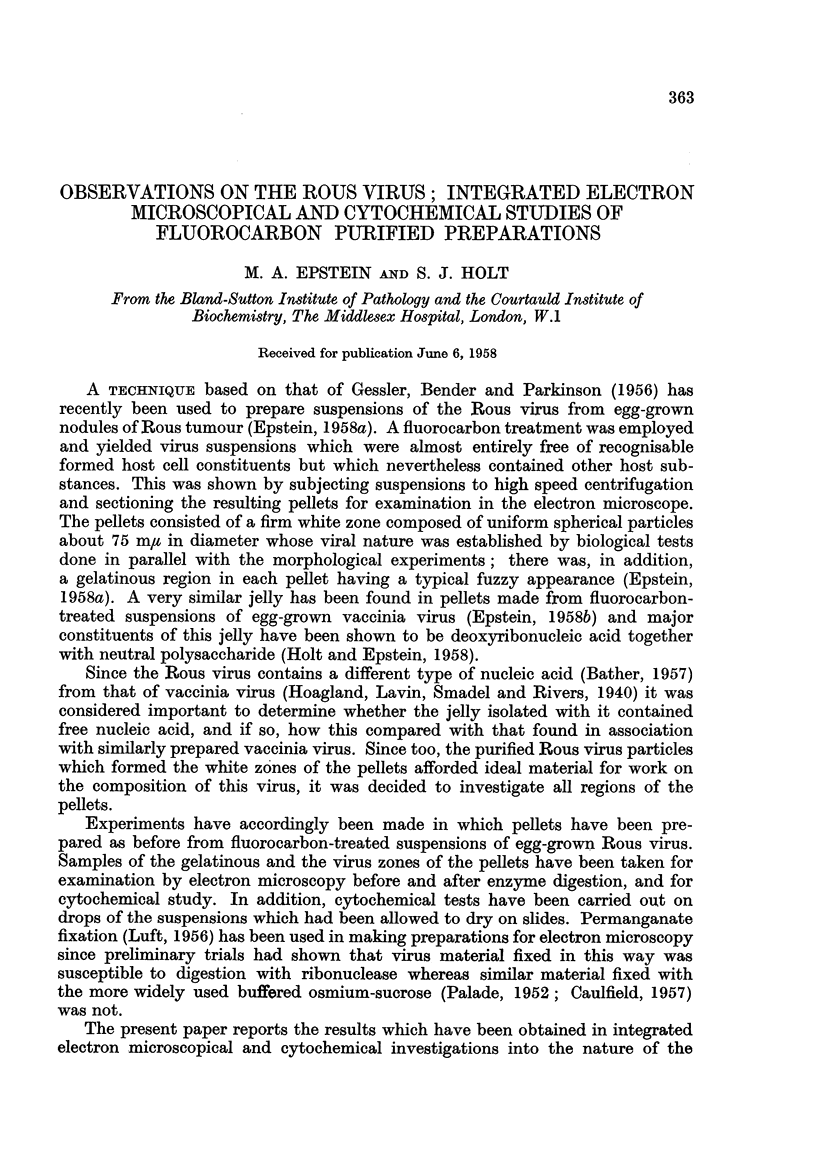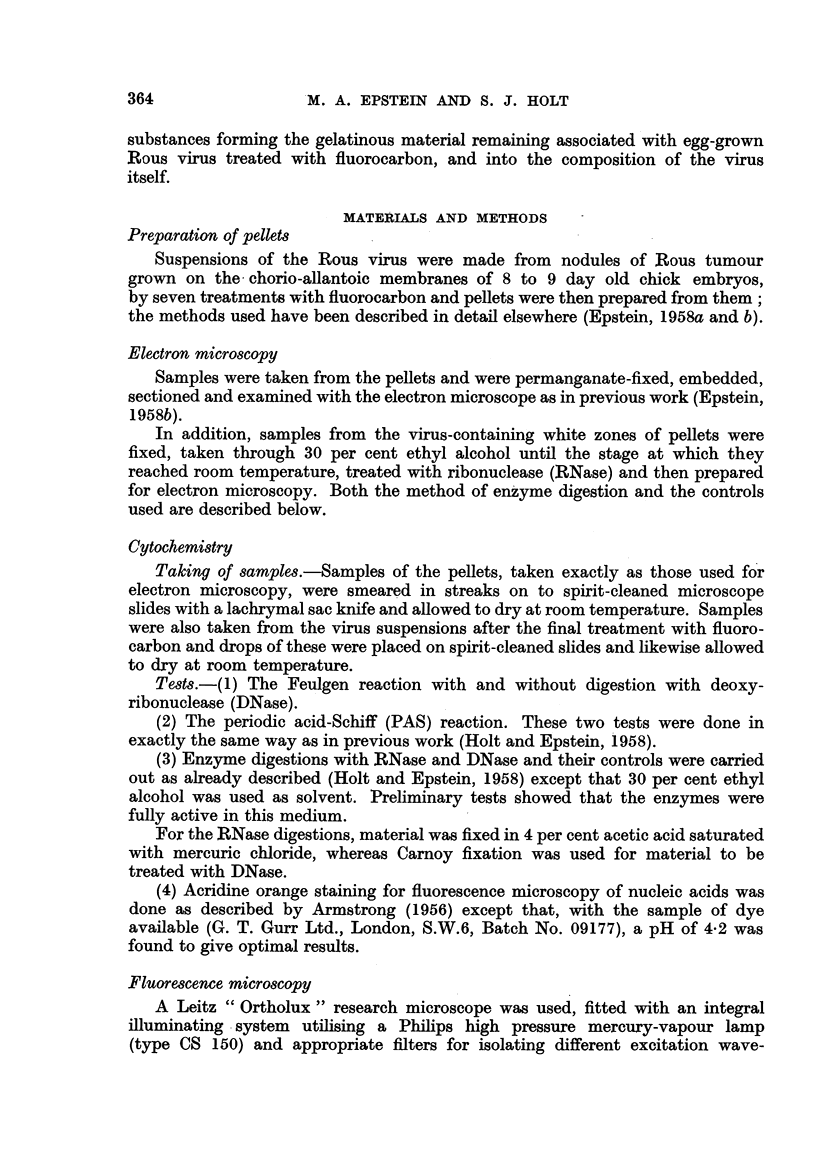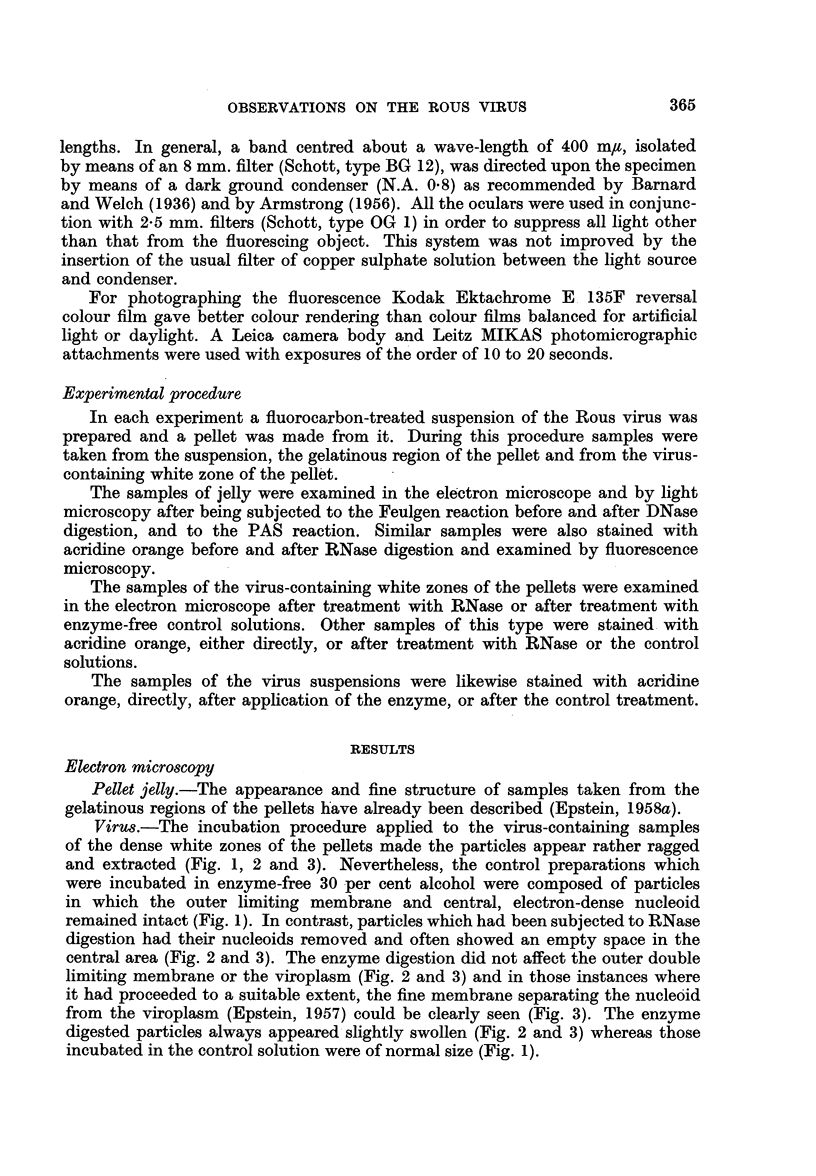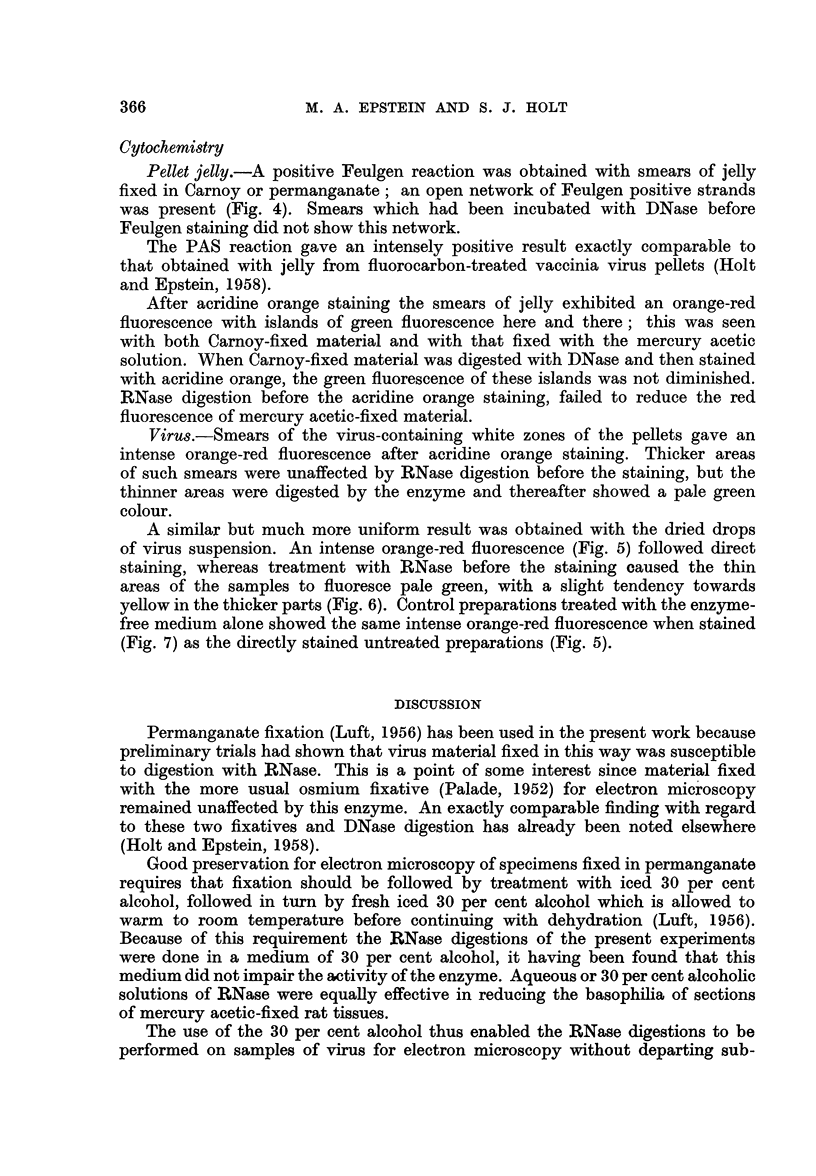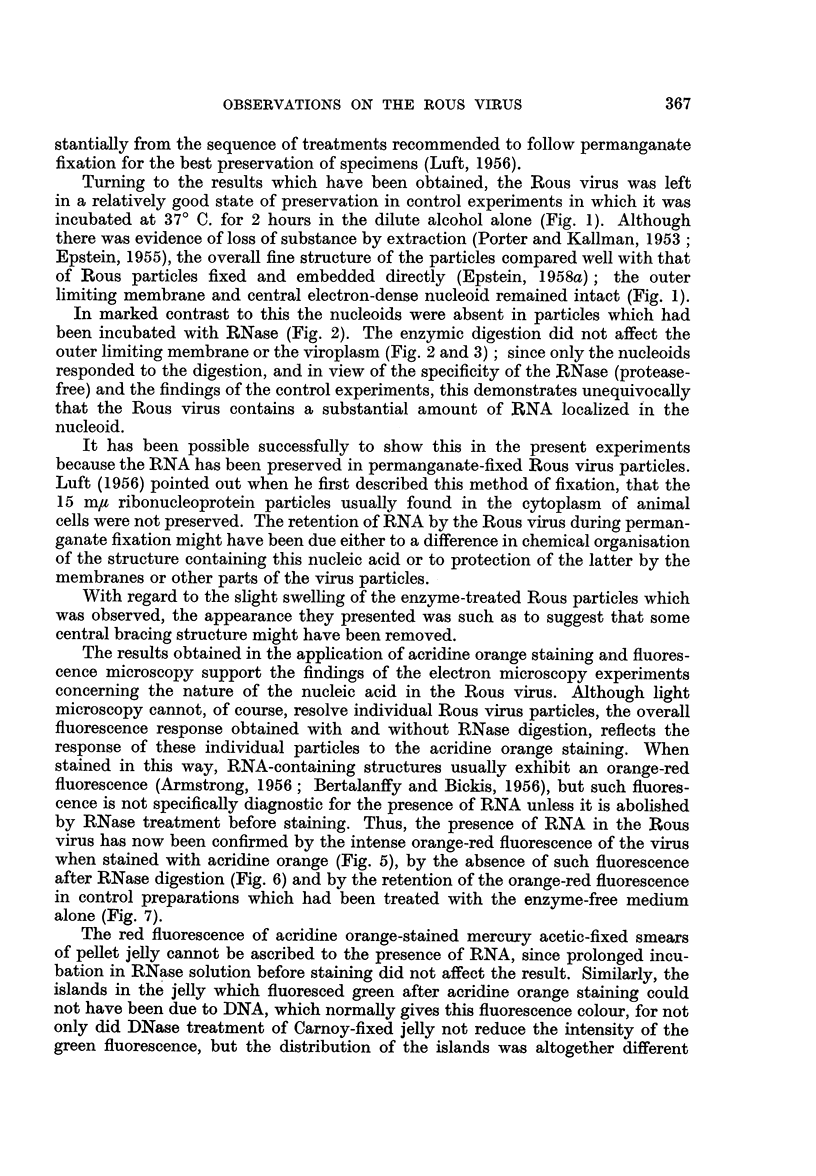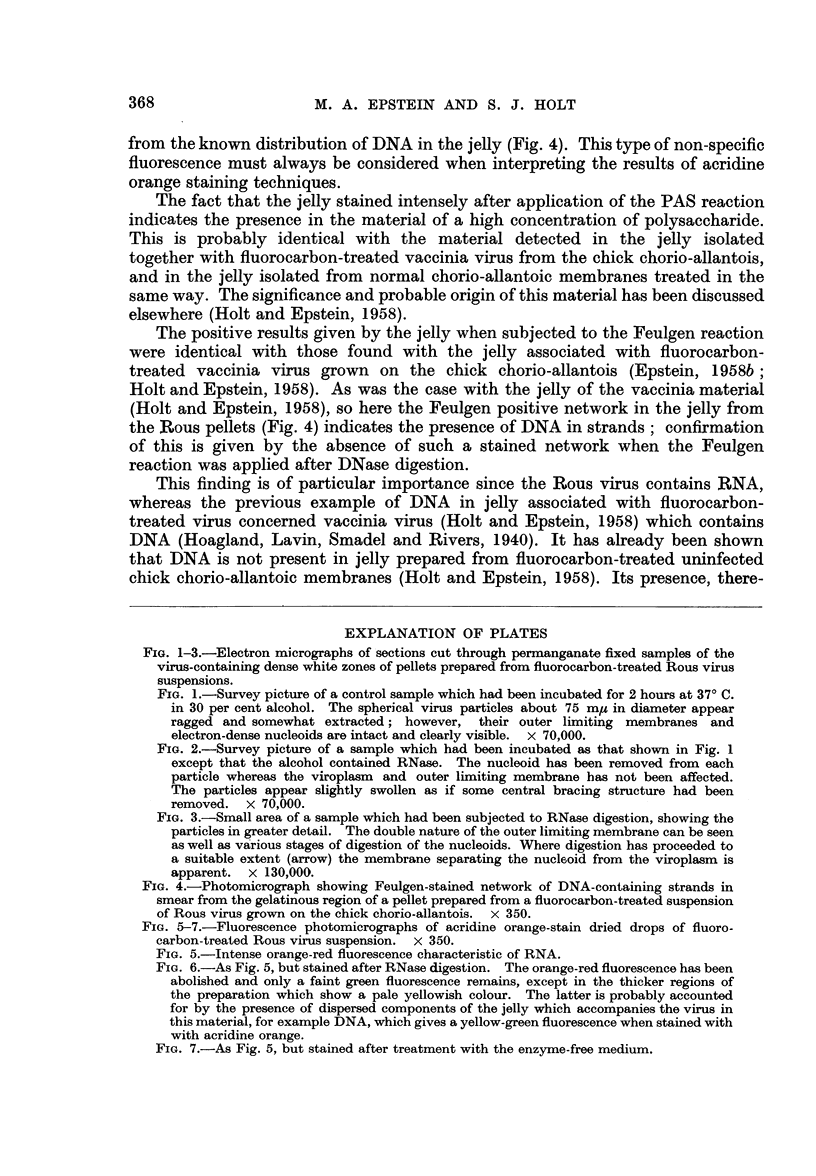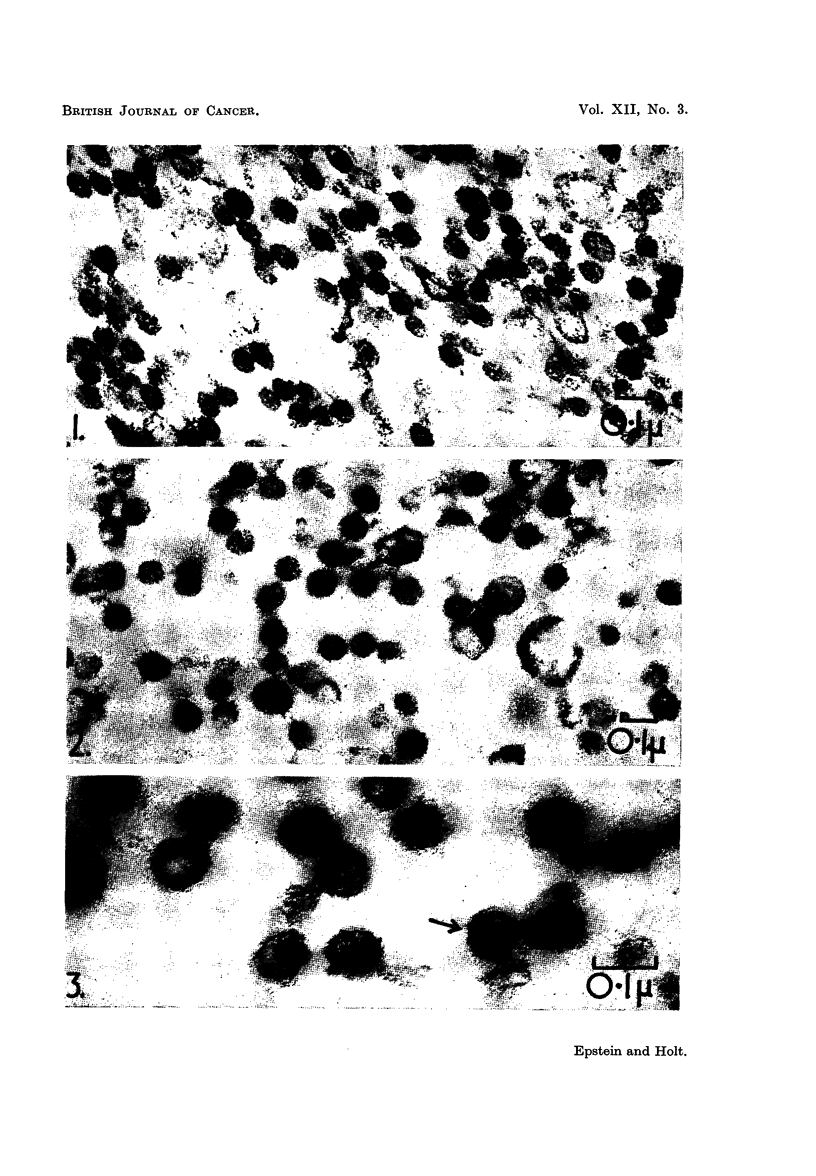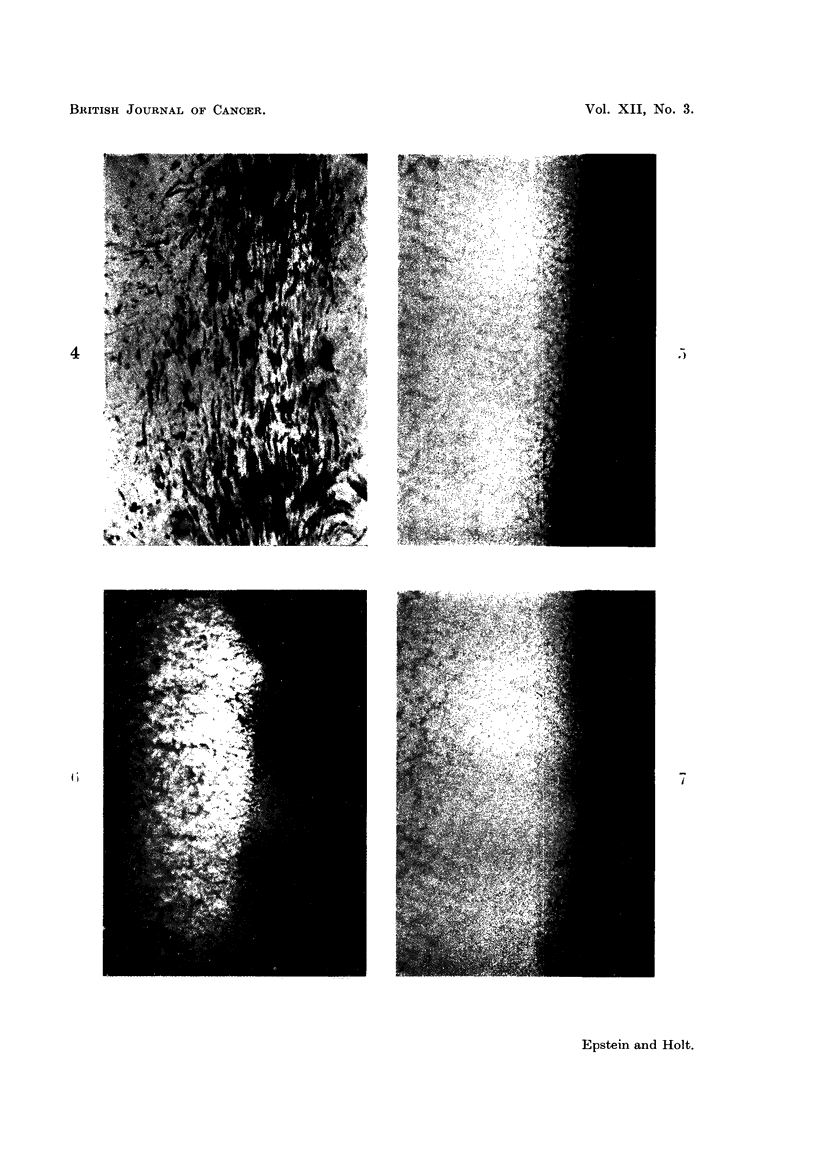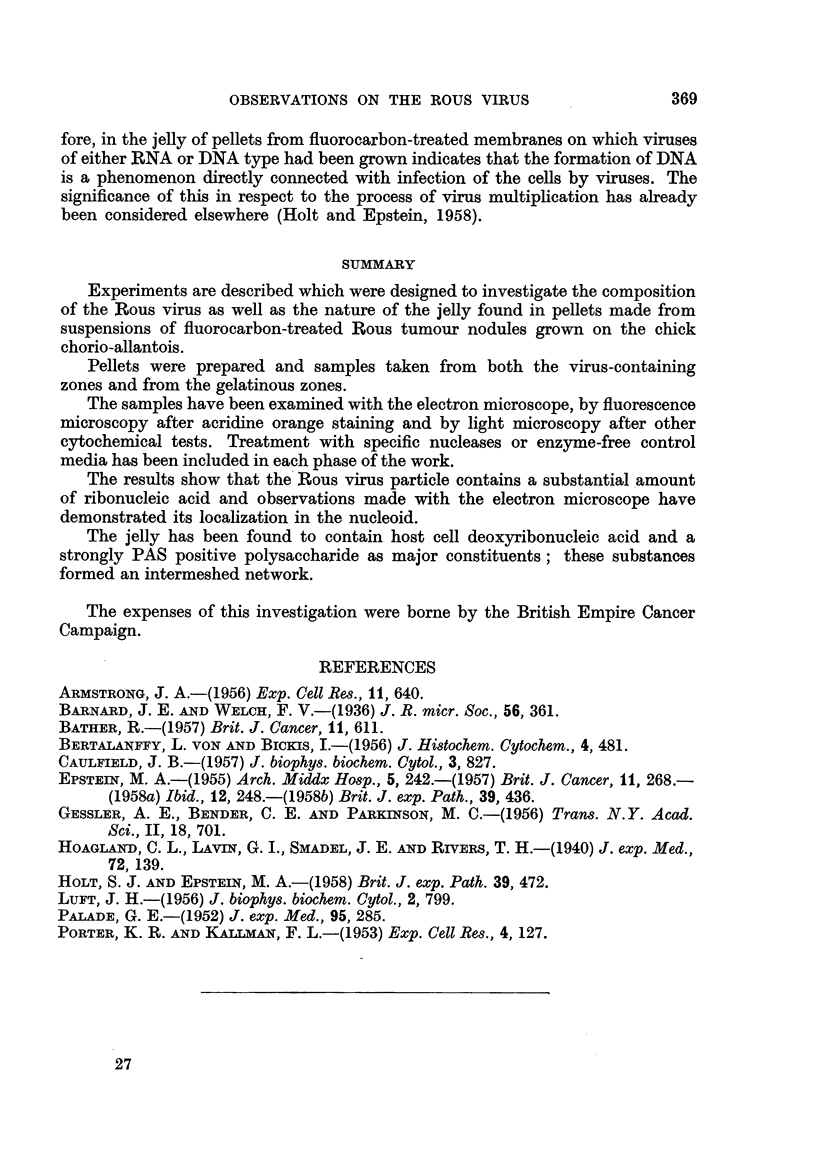# Observations on the Rous Virus; Integrated Electron Microscopical and Cytochemical Studies of Fluorocarbon Purified Preparations

**DOI:** 10.1038/bjc.1958.45

**Published:** 1958-09

**Authors:** M. A. Epstein, S. J. Holt

## Abstract

**Images:**


### OBSERVATIONS ON THE ROUS VIRUS ; INTEGRATED ELECTRON MICROSCOPICAL AND CYTOCHEMICAL STUDIES OF FLUOROCARBON PURIFIED PREPARATIONS

M. A. EPSTEIN AND S. J. HOLT

*From the Bland-Sutton Institute of Pathology and the Courtauld Institute of Biochemistry, The Middlesex Hospital, London, W.1*

Received for publication June 6, 1958

A TECHNIQUE based on that of Gessler, Bender and Parkinson (1956) has recently been used to prepare suspensions of the Rous virus from egg-grown nodules of Rous tumour (Epstein, 1958*a*). A fluorocarbon treatment was employed and yielded virus suspensions which were almost entirely free of recognisable formed host cell constituents but which nevertheless contained other host substances. This was shown by subjecting suspensions to high speed centrifugation and sectioning the resulting pellets for examination in the electron microscope. The pellets consisted of a firm white zone composed of uniform spherical particles about 75 m$\mu$ in diameter whose viral nature was established by biological tests done in parallel with the morphological experiments ; there was, in addition, a gelatinous region in each pellet having a typical fuzzy appearance (Epstein, 1958*a*). A very similar jelly has been found in pellets made from fluorocarbon-treated suspensions of egg-grown vaccinia virus (Epstein, 1958*b*) and major constituents of this jelly have been shown to be deoxyribonucleic acid together with neutral polysaccharide (Holt and Epstein, 1958).

Since the Rous virus contains a different type of nucleic acid (Bather, 1957) from that of vaccinia virus (Hoagland, Lavin, Smadel and Rivers, 1940) it was considered important to determine whether the jelly isolated with it contained free nucleic acid, and if so, how this compared with that found in association with similarly prepared vaccinia virus. Since too, the purified Rous virus particles which formed the white zones of the pellets afforded ideal material for work on the composition of this virus, it was decided to investigate all regions of the pellets.

Experiments have accordingly been made in which pellets have been prepared as before from fluorocarbon-treated suspensions of egg-grown Rous virus. Samples of the gelatinous and the virus zones of the pellets have been taken for examination by electron microscopy before and after enzyme digestion, and for cytochemical study. In addition, cytochemical tests have been carried out on drops of the suspensions which had been allowed to dry on slides. Permanganate fixation (Luft, 1956) has been used in making preparations for electron microscopy since preliminary trials had shown that virus material fixed in this way was susceptible to digestion with ribonuclease whereas similar material fixed with the more widely used buffered osmium-sucrose (Palade, 1952 ; Caulfield, 1957) was not.

The present paper reports the results which have been obtained in integrated electron microscopical and cytochemical investigations into the nature of the substances forming the gelatinous material remaining associated with egg-grown Rous virus treated with fluorocarbon, and into the composition of the virus itself.

### MATERIALS AND METHODS

### *Preparation of pellets*

Suspensions of the Rous virus were made from nodules of Rous tumour grown on the chorio-allantoic membranes of 8 to 9 day old chick embryos, by seven treatments with fluorocarbon and pellets were then prepared from them ; the methods used have been described in detail elsewhere (Epstein, 1958*a* and *b*).

### *Electron microscopy*

Samples were taken from the pellets and were permanganate-fixed, embedded, sectioned and examined with the electron microscope as in previous work (Epstein, 1958*b*).

In addition, samples from the virus-containing white zones of pellets were fixed, taken through 30 per cent ethyl alcohol until the stage at which they reached room temperature, treated with ribonuclease (RNase) and then prepared for electron microscopy. Both the method of enzyme digestion and the controls used are described below.

### *Cytochemistry*

*Taking of samples.*—Samples of the pellets, taken exactly as those used for electron microscopy, were smeared in streaks on to spirit-cleaned microscope slides with a lachrymal sac knife and allowed to dry at room temperature. Samples were also taken from the virus suspensions after the final treatment with fluorocarbon and drops of these were placed on spirit-cleaned slides and likewise allowed to dry at room temperature.

*Tests.*—(1) The Feulgen reaction with and without digestion with deoxyribonuclease (DNase).

(2) The periodic acid-Schiff (PAS) reaction. These two tests were done in exactly the same way as in previous work (Holt and Epstein, 1958).

(3) Enzyme digestions with RNase and DNase and their controls were carried out as already described (Holt and Epstein, 1958) except that 30 per cent ethyl alcohol was used as solvent. Preliminary tests showed that the enzymes were fully active in this medium.

For the RNase digestions, material was fixed in 4 per cent acetic acid saturated with mercuric chloride, whereas Carnoy fixation was used for material to be treated with DNase.

(4) Acridine orange staining for fluorescence microscopy of nucleic acids was done as described by Armstrong (1956) except that, with the sample of dye available (G. T. Gurr Ltd., London, S.W.6, Batch No. 09177), a pH of 4·2 was found to give optimal results.

### *Fluorescence microscopy*

A Leitz " Ortholux " research microscope was used, fitted with an integral illuminating system utilising a Philips high pressure mercury-vapour lamp (type CS 150) and appropriate filters for isolating different excitation wavelengths. In general, a band centred about a wave-length of 400 m$\mu$, isolated by means of an 8 mm. filter (Schott, type BG 12), was directed upon the specimen by means of a dark ground condenser (N.A. 0·8) as recommended by Barnard and Welch (1936) and by Armstrong (1956). All the oculars were used in conjunction with 2·5 mm. filters (Schott, type OG 1) in order to suppress all light other than that from the fluorescing object. This system was not improved by the insertion of the usual filter of copper sulphate solution between the light source and condenser.

For photographing the fluorescence Kodak Ektachrome E 135F reversal colour film gave better colour rendering than colour films balanced for artificial light or daylight. A Leica camera body and Leitz MIKAS photomicrographic attachments were used with exposures of the order of 10 to 20 seconds.

*Experimental procedure*

In each experiment a fluorocarbon-treated suspension of the Rous virus was prepared and a pellet was made from it. During this procedure samples were taken from the suspension, the gelatinous region of the pellet and from the virus-containing white zone of the pellet.

The samples of jelly were examined in the electron microscope and by light microscopy after being subjected to the Feulgen reaction before and after DNase digestion, and to the PAS reaction. Similar samples were also stained with acridine orange before and after RNase digestion and examined by fluorescence microscopy.

The samples of the virus-containing white zones of the pellets were examined in the electron microscope after treatment with RNase or after treatment with enzyme-free control solutions. Other samples of this type were stained with acridine orange, either directly, or after treatment with RNase or the control solutions.

The samples of the virus suspensions were likewise stained with acridine orange, directly, after application of the enzyme, or after the control treatment.

### RESULTS

*Electron microscopy*

*Pellet jelly.*—The appearance and fine structure of samples taken from the gelatinous regions of the pellets have already been described (Epstein, 1958*a*).

*Virus.*—The incubation procedure applied to the virus-containing samples of the dense white zones of the pellets made the particles appear rather ragged and extracted (Fig. 1, 2 and 3). Nevertheless, the control preparations which were incubated in enzyme-free 30 per cent alcohol were composed of particles in which the outer limiting membrane and central, electron-dense nucleoid remained intact (Fig. 1). In contrast, particles which had been subjected to RNase digestion had their nucleoids removed and often showed an empty space in the central area (Fig. 2 and 3). The enzyme digestion did not affect the outer double limiting membrane or the viroplasm (Fig. 2 and 3) and in those instances where it had proceeded to a suitable extent, the fine membrane separating the nucleöid from the viroplasm (Epstein, 1957) could be clearly seen (Fig. 3). The enzyme digested particles always appeared slightly swollen (Fig. 2 and 3) whereas those incubated in the control solution were of normal size (Fig. 1).

*Cytochemistry*

*Pellet jelly.*—A positive Feulgen reaction was obtained with smears of jelly fixed in Carnoy or permanganate ; an open network of Feulgen positive strands was present (Fig. 4). Smears which had been incubated with DNase before Feulgen staining did not show this network.

The PAS reaction gave an intensely positive result exactly comparable to that obtained with jelly from fluorocarbon-treated vaccinia virus pellets (Holt and Epstein, 1958).

After acridine orange staining the smears of jelly exhibited an orange-red fluorescence with islands of green fluorescence here and there ; this was seen with both Carnoy-fixed material and with that fixed with the mercury acetic solution. When Carnoy-fixed material was digested with DNase and then stained with acridine orange, the green fluorescence of these islands was not diminished. RNase digestion before the acridine orange staining, failed to reduce the red fluorescence of mercury acetic-fixed material.

*Virus.*—Smears of the virus-containing white zones of the pellets gave an intense orange-red fluorescence after acridine orange staining. Thicker areas of such smears were unaffected by RNase digestion before the staining, but the thinner areas were digested by the enzyme and thereafter showed a pale green colour.

A similar but much more uniform result was obtained with the dried drops of virus suspension. An intense orange-red fluorescence (Fig. 5) followed direct staining, whereas treatment with RNase before the staining caused the thin areas of the samples to fluoresce pale green, with a slight tendency towards yellow in the thicker parts (Fig. 6). Control preparations treated with the enzyme-free medium alone showed the same intense orange-red fluorescence when stained (Fig. 7) as the directly stained untreated preparations (Fig. 5).

### DISCUSSION

Permanganate fixation (Luft, 1956) has been used in the present work because preliminary trials had shown that virus material fixed in this way was susceptible to digestion with RNase. This is a point of some interest since material fixed with the more usual osmium fixative (Palade, 1952) for electron microscopy remained unaffected by this enzyme. An exactly comparable finding with regard to these two fixatives and DNase digestion has already been noted elsewhere (Holt and Epstein, 1958).

Good preservation for electron microscopy of specimens fixed in permanganate requires that fixation should be followed by treatment with iced 30 per cent alcohol, followed in turn by fresh iced 30 per cent alcohol which is allowed to warm to room temperature before continuing with dehydration (Luft, 1956). Because of this requirement the RNase digestions of the present experiments were done in a medium of 30 per cent alcohol, it having been found that this medium did not impair the activity of the enzyme. Aqueous or 30 per cent alcoholic solutions of RNase were equally effective in reducing the basophilia of sections of mercury acetic-fixed rat tissues.

The use of the 30 per cent alcohol thus enabled the RNase digestions to be performed on samples of virus for electron microscopy without departing substantially from the sequence of treatments recommended to follow permanganate fixation for the best preservation of specimens (Luft, 1956).

Turning to the results which have been obtained, the Rous virus was left in a relatively good state of preservation in control experiments in which it was incubated at 37° C. for 2 hours in the dilute alcohol alone (Fig. 1). Although there was evidence of loss of substance by extraction (Porter and Kallman, 1953 ; Epstein, 1955), the overall fine structure of the particles compared well with that of Rous particles fixed and embedded directly (Epstein, 1958*a*) ; the outer limiting membrane and central electron-dense nucleoid remained intact (Fig. 1).

In marked contrast to this the nucleoids were absent in particles which had been incubated with RNase (Fig. 2). The enzymic digestion did not affect the outer limiting membrane or the viroplasm (Fig. 2 and 3) ; since only the nucleoids responded to the digestion, and in view of the specificity of the RNase (protease-free) and the findings of the control experiments, this demonstrates unequivocally that the Rous virus contains a substantial amount of RNA localized in the nucleoid.

It has been possible successfully to show this in the present experiments because the RNA has been preserved in permanganate-fixed Rous virus particles. Luft (1956) pointed out when he first described this method of fixation, that the 15 m$\mu$ ribonucleoprotein particles usually found in the cytoplasm of animal cells were not preserved. The retention of RNA by the Rous virus during permanganate fixation might have been due either to a difference in chemical organisation of the structure containing this nucleic acid or to protection of the latter by the membranes or other parts of the virus particles.

With regard to the slight swelling of the enzyme-treated Rous particles which was observed, the appearance they presented was such as to suggest that some central bracing structure might have been removed.

The results obtained in the application of acridine orange staining and fluorescence microscopy support the findings of the electron microscopy experiments concerning the nature of the nucleic acid in the Rous virus. Although light microscopy cannot, of course, resolve individual Rous virus particles, the overall fluorescence response obtained with and without RNase digestion, reflects the response of these individual particles to the acridine orange staining. When stained in this way, RNA-containing structures usually exhibit an orange-red fluorescence (Armstrong, 1956 ; Bertalanffy and Bickis, 1956), but such fluorescence is not specifically diagnostic for the presence of RNA unless it is abolished by RNase treatment before staining. Thus, the presence of RNA in the Rous virus has now been confirmed by the intense orange-red fluorescence of the virus when stained with acridine orange (Fig. 5), by the absence of such fluorescence after RNase digestion (Fig. 6) and by the retention of the orange-red fluorescence in control preparations which had been treated with the enzyme-free medium alone (Fig. 7).

The red fluorescence of acridine orange-stained mercury acetic-fixed smears of pellet jelly cannot be ascribed to the presence of RNA, since prolonged incubation in RNase solution before staining did not affect the result. Similarly, the islands in the jelly which fluoresced green after acridine orange staining could not have been due to DNA, which normally gives this fluorescence colour, for not only did DNase treatment of Carnoy-fixed jelly not reduce the intensity of the green fluorescence, but the distribution of the islands was altogether different from the known distribution of DNA in the jelly (Fig. 4). This type of non-specific fluorescence must always be considered when interpreting the results of acridine orange staining techniques.

The fact that the jelly stained intensely after application of the PAS reaction indicates the presence in the material of a high concentration of polysaccharide. This is probably identical with the material detected in the jelly isolated together with fluorocarbon-treated vaccinia virus from the chick chorio-allantois, and in the jelly isolated from normal chorio-allantoic membranes treated in the same way. The significance and probable origin of this material has been discussed elsewhere (Holt and Epstein, 1958).

The positive results given by the jelly when subjected to the Feulgen reaction were identical with those found with the jelly associated with fluorocarbon-treated vaccinia virus grown on the chick chorio-allantois (Epstein, 1958*b*; Holt and Epstein, 1958). As was the case with the jelly of the vaccinia material (Holt and Epstein, 1958), so here the Feulgen positive network in the jelly from the Rous pellets (Fig. 4) indicates the presence of DNA in strands; confirmation of this is given by the absence of such a stained network when the Feulgen reaction was applied after DNase digestion.

This finding is of particular importance since the Rous virus contains RNA, whereas the previous example of DNA in jelly associated with fluorocarbon-treated virus concerned vaccinia virus (Holt and Epstein, 1958) which contains DNA (Hoagland, Lavin, Smadel and Rivers, 1940). It has already been shown that DNA is not present in jelly prepared from fluorocarbon-treated uninfected chick chorio-allantoic membranes (Holt and Epstein, 1958). Its presence, there-

---

EXPLANATION OF PLATES

FIG. 1–3.—Electron micrographs of sections cut through permanganate fixed samples of the virus-containing dense white zones of pellets prepared from fluorocarbon-treated Rous virus suspensions.

FIG. 1.—Survey picture of a control sample which had been incubated for 2 hours at 37° C. in 30 per cent alcohol. The spherical virus particles about 75 m$\mu$ in diameter appear ragged and somewhat extracted; however, their outer limiting membranes and electron-dense nucleoids are intact and clearly visible. $\times$ 70,000.

FIG. 2.—Survey picture of a sample which had been incubated as that shown in Fig. 1 except that the alcohol contained RNase. The nucleoid has been removed from each particle whereas the viroplasm and outer limiting membrane has not been affected. The particles appear slightly swollen as if some central bracing structure had been removed. $\times$ 70,000.

FIG. 3.—Small area of a sample which had been subjected to RNase digestion, showing the particles in greater detail. The double nature of the outer limiting membrane can be seen as well as various stages of digestion of the nucleoids. Where digestion has proceeded to a suitable extent (arrow) the membrane separating the nucleoid from the viroplasm is apparent. $\times$ 130,000.

FIG. 4.—Photomicrograph showing Feulgen-stained network of DNA-containing strands in smear from the gelatinous region of a pellet prepared from a fluorocarbon-treated suspension of Rous virus grown on the chick chorio-allantois. $\times$ 350.

FIG. 5–7.—Fluorescence photomicrographs of acridine orange-stain dried drops of fluorocarbon-treated Rous virus suspension. $\times$ 350.

FIG. 5.—Intense orange-red fluorescence characteristic of RNA.

FIG. 6.—As Fig. 5, but stained after RNase digestion. The orange-red fluorescence has been abolished and only a faint green fluorescence remains, except in the thicker regions of the preparation which show a pale yellowish colour. The latter is probably accounted for by the presence of dispersed components of the jelly which accompanies the virus in this material, for example DNA, which gives a yellow-green fluorescence when stained with with acridine orange.

FIG. 7.—As Fig. 5, but stained after treatment with the enzyme-free medium.

1.
0·1 μ

2.
0·1 μ

3.
0·1 μ

4

5

6

7

Epstein and Holt.

fore, in the jelly of pellets from fluorocarbon-treated membranes on which viruses of either RNA or DNA type had been grown indicates that the formation of DNA is a phenomenon directly connected with infection of the cells by viruses. The significance of this in respect to the process of virus multiplication has already been considered elsewhere (Holt and Epstein, 1958).

### SUMMARY

Experiments are described which were designed to investigate the composition of the Rous virus as well as the nature of the jelly found in pellets made from suspensions of fluorocarbon-treated Rous tumour nodules grown on the chick chorio-allantois.

Pellets were prepared and samples taken from both the virus-containing zones and from the gelatinous zones.

The samples have been examined with the electron microscope, by fluorescence microscopy after acridine orange staining and by light microscopy after other cytochemical tests. Treatment with specific nucleases or enzyme-free control media has been included in each phase of the work.

The results show that the Rous virus particle contains a substantial amount of ribonucleic acid and observations made with the electron microscope have demonstrated its localization in the nucleoid.

The jelly has been found to contain host cell deoxyribonucleic acid and a strongly PAS positive polysaccharide as major constituents ; these substances formed an intermeshed network.

The expenses of this investigation were borne by the British Empire Cancer Campaign.

### REFERENCES

Armstrong, J. A.—(1956) *Exp. Cell Res.*, **11**, 640.
Barnard, J. E. and Welch, F. V.—(1936) *J. R. micr. Soc.*, **56**, 361.
Bather, R.—(1957) *Brit. J. Cancer*, **11**, 611.
Bertalanffy, L. von and Bickis, I.—(1956) *J. Histochem. Cytochem.*, **4**, 481.
Caulfield, J. B.—(1957) *J. biophys. biochem. Cytol.*, **3**, 827.
Epstein, M. A.—(1955) *Arch. Middx Hosp.*, **5**, 242.—(1957) *Brit. J. Cancer*, **11**, 268.—(1958*a*) *Ibid.*, **12**, 248.—(1958*b*) *Brit. J. exp. Path.*, **39**, 436.
Gessler, A. E., Bender, C. E. and Parkinson, M. C.—(1956) *Trans. N.Y. Acad. Sci.*, II, 18, 701.
Hoagland, C. L., Lavin, G. I., Smadel, J. E. and Rivers, T. H.—(1940) *J. exp. Med.*, **72**, 139.
Holt, S. J. and Epstein, M. A.—(1958) *Brit. J. exp. Path.* **39**, 472.
Luft, J. H.—(1956) *J. biophys. biochem. Cytol.*, **2**, 799.
Palade, G. E.—(1952) *J. exp. Med.*, **95**, 285.
Porter, K. R. and Kallman, F. L.—(1953) *Exp. Cell Res.*, **4**, 127.